# Statistical regularities bias overt attention

**DOI:** 10.3758/s13414-019-01708-5

**Published:** 2019-03-27

**Authors:** Benchi Wang, Iliana Samara, Jan Theeuwes

**Affiliations:** Department of Experimental and Applied Psychology, Vrije Universiteit Amsterdam, Van der Boechorststraat 1, 1081 BT Amsterdam, The Netherlands

**Keywords:** Attentional capture, Statistical regularities, Oculomotor suppression, Rapid disengagement hypothesis, Spatial priority map

## Abstract

A previous study employing the additional singleton paradigm showed that a singleton distractor that appeared more often in one specific location interfered less with target search than when it appeared at any other location. These findings suggested that through statistical learning the location that was likely to contain a distractor was suppressed relative to all other locations. Even though feasible, it is also possible that this effect is due to faster disengagement of attention from the high-probability distractor location. The present study tested this hypothesis using a variant of the additional singleton task adapted for eye tracking in which observers made a speeded saccade to a shape singleton and gave a manual response. The singleton distractor was presented more often at one location than all other locations. Consistent with the suppression hypothesis, we found that fewer saccades landed at the high-probability distractor location than any other location. Also, when a target appeared at the high-probability location, saccade latencies towards the target were higher than latencies towards the target when it was presented at other locations. Furthermore, in addition to suppression, we also found evidence for faster disengagement from the high-probability distractor location than the low-probability distractor location; however, this effect was relatively small. The current findings support the notion that through statistical learning plasticity is induced in the spatial priority map of attentional selection so that the high-probability distractor location is suppressed compared to any other location.

## Introduction

To successfully navigate our environment, the selection and suppression of visual information is of central importance for the visual system. Selective attention allows us to focus on particular items in the display while filtering irrelevant information (Jonides & Yantis, 1988; Yantis & Jonides, 1990). Traditionally, selective attention is assumed to be guided either by the goals of the observer (top-down control; Egeth & Yantis, 1997; Folk, Remington, & Johnston, 1992) or the properties of the stimulus (bottom-up control; Theeuwes, 2010; Yantis & Egeth, 1999). Despite this well-documented dichotomy of attentional control, another source of attentional bias was recently proposed, namely "selection history," which reflects the influence of prior selection experiences on current selection. Selection history encompasses sources of bias that cannot be explained by the traditional bottom-up, top-down dichotomy (Awh, Belopolsky, & Theeuwes, 2012; see Failing & Theeuwes, 2018 for a recent review), such as inter-trial priming (Maljkovic & Nakayama, 1994; Tipper, 1985); biases due to reward (Bucker & Theeuwes, 2017; Chelazzi, Perlato, Santandrea, & Della Libera, 2013; Della Libera, Perlato, & Chelazzi, 2011; Failing, Nissens, Pearson, Le Pelley, & Theeuwes, 2015; Preciado, Munneke, & Theeuwes, 2017a); and threat (Nissens, Failing, & Theeuwes, 2017; Preciado, Munneke, & Theeuwes, 2017b; Schmidt, Belopolsky, & Theeuwes, 2015).

Besides the aforementioned sources of bias, recent studies have revealed that selection history due to statistical learning (SL) can result in lingering selection biases (Ferrante et al., 2018; Wang & Theeuwes, 2018a, 2018b). SL is defined as the ability to extract events that co-occur in our environment and utilize this learned covariance to implicitly deploy our attentional resources in an efficient manner (Schapiro & Turk-Browne, 2015). Studies examining the effect of SL on attention have typically manipulated the spatial or temporal distribution of the target within experimental paradigms (Chun & Jiang, 1998, 1999; Geng & Behrmann, 2002, 2005; Miller, 1988; Shaw & Shaw, 1977). Findings from these studies demonstrate that observers respond faster to targets appearing systematically at a specific location. Furthermore, more eye movements land on the target when it appears at a high-probability location than other locations on the display (Walthew & Gilchrist, 2006). Even though these studies show that people easily pick up on statistical regularities concerning the location of the target, this may not be an unexpected finding given that the target is highly relevant for the task: strategic prioritization of likely target locations leads to improved performance. Moreover, since the pioneering cueing studies of Posner (1980), it is well established that observers can strategically allocate their attention to locations in which the target is more likely to appear.

Even though it may not be surprising that people can learn to attend target objects that are relevant for the task at hand, more recent studies demonstrated that people also learn statistical regularities regarding task-irrelevant stimuli such as salient distractors (Wang & Theeuwes, 2018a, 2018b). Obviously, avoiding distraction by salient irrelevant distractors is important for accomplishing daily tasks. Wang and Theeuwes’ studies showed that systematic manipulation of the distractor location decreased the distractor interference effects (see also Ferrante et al., 2018; Goschy, Bakos, Müller, & Zehetleitner, 2014). Wang and Theeuwes (2018a), for example, employed the additional singleton paradigm (Theeuwes, 1991, 1992) where observers search for a shape singleton target while ignoring a salient color distractor singleton. Importantly, the salient distractor was displayed with a higher probability at a specific location of the visual field. Their results demonstrated that when the salient distractor was displayed at the high-probability location, there was less interference than when the distractor was displayed at a low-probability location. Furthermore, target detection was impaired when the target appeared at the high-probability distractor location. Similar to other accounts (Wang & Theeuwes, 2018b; Zhao, Al-Aidroos, & Turk-Browne, 2013), despite the fact that observers exhibited biases against directing attention to the high-probability distractor location, they could not report the high-probability distractor location.

The reduced response time (RT) interference effect observed by Wang and Theeuwes (2018a) was suggested to be due to the suppression of the high-probability distractor location. Even though this may seem reasonable, it should be noted that alternative interpretations are possible. One such account is that observers learn to quickly disengage attention from the high-probability distractor location and are relatively slow in disengaging attention from the low-probability distractor location, as it has been shown that observers are faster to disengage from expected than unexpected distractors (Brockmole & Boot, 2009). Faster disengagement from the high-probability distractor location would also result in a reduction of the interference effect compared to the low-probability locations (Theeuwes, 2010). This idea of rapid disengagement was introduced by Theeuwes and colleagues (Theeuwes, Kramer, Hahn, Irwin, & Zelinsky, 1999; see also Theeuwes, 2010) to explain why, in some paradigms, it may seem that abrupt onsets do not capture attention. The assumption is that attention is initially captured by a salient distractor but is immediately released when it is clear that the object selected is not the target. Rapid disengagement is assumed to be driven by top-down control, reflecting post-selection processes (Godijn & Theeuwes, 2002; Mulckhuyse, Van der Stigchel, & Theeuwes, 2009; Schreij, Theeuwes, & Olivers, 2010; Theeuwes, 2010; Theeuwes, De Vries, & Godijn, 2003) as opposed to oculomotor capture, which is driven by bottom-up processes (Born, Kerzel, & Theeuwes, 2011). Therefore, speeded disengagement from the high-probability distractor location would imply the involvement of top-down processes in reducing the reported interference effects, rather than attentional biases resulting from selection history. The present study aimed to investigate whether the reduced interference effect that is observed when the distractors are presented at a high-probability location is the result of attentional suppression of the high-probability distractor location or alternatively the result of speeded disengagement from the high-probability distractor location. One way to examine this is to examine overt attentional selection (i.e., eye movements), as the attentional and oculomotor systems are associated (Deubel & Schneider, 1996; Godijn & Theeuwes, 2002; McPeek, Maljkovic, & Nakayama, 1999; Rizzolatti, Riggio, Dascola, & Umiltá, 1987) and similar capture effects reported with covert attention have also been reported with overt selection (Theeuwes et al., 2003, 1999). Therefore, we expected that if the high-probability distractor location is suppressed, fewer first saccades should land on the salient distractor when it appears at the high-probability distractor location than the low-probability distractor locations (oculomotor suppression effect). If reduced interference is due to rapid disengagement, then fixations on the distractor should be shorter when it is displayed at the high-probability distractor location than when displayed at a low-probability distractor location.

## Methods

### Participants

We recruited 16 participants (14 women, *M* age = 20.6 years, *SD* = 2.8) with reported normal or corrected-to-normal vision and normal color vision. Sample size was predetermined based on the significant difference between high-probability location and low-probability location in Wang and Theeuwes (2018a), with an effect size of 1.83. With 16 subjects and alpha = .001, power for the critical effect should be > 0.99.

All participants gave informed consent in accordance with to the Declaration of Helsinki. The study received approval by the Ethics Committee of the Vrije Universiteit Amsterdam and participants received either monetary compensation (8 €/h) or course credits.

### Apparatus and stimuli

The experimental task was written in a custom Python script. Stimuli were presented on a

22-in. monitor with a resolution of 1,280 × 1,024 and a refresh rate of 120 Hz. Participants were seated in individual dimly-lit, soundproof cabins at approximately 70 cm from the monitor with their head resting on a chin rest for the duration of the task. Eye movements were recorded with an Eyelink 1000 eye tracker (sampling rate 1,000 Hz).

**Fig. 1:**
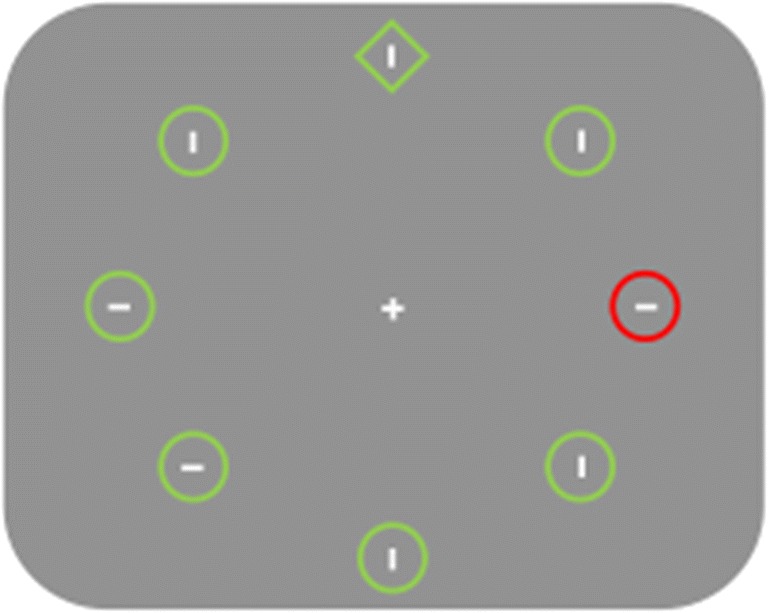
A typical experimental display in the variant of the additional singleton task

Figure 1 depicts the experimental search display. All stimuli were displayed on the radius of an imaginary circle (6.8°), centered at the fixation cross (0.85° × 0.85°), against a gray background. The visual search array consisted of eight stimuli: either one circle (radius of 0.85°) amongst seven diamonds (subtended 1.7° ×1.7°) or vice versa. All stimuli were outline shapes presented in either red or green and contained either a vertical or a horizontal line segment.

### Procedure and design

At the start of each trial, observers performed a self-paced drift correction. Subsequently, observers were required to fixate for 500 ms on a centrally presented fixation cross. Then, the search array appeared for 3,000 ms or until a response was given. Participants identified the shape singleton (either a diamond amongst distractor circles or vice versa) and reported the orientation of the line segment (vertical or horizontal) appearing within the shape by pressing the corresponding keyboard key (left-arrow key for horizontal, up-arrow key for vertical). Every trial finished with an inter-trial interval (ITI) of 500–750 ms.

In two-thirds of the trials, one of the non-target elements in the search display was a uniquely colored singleton (green or red with equal chance, henceforth called the distractor). The colored distractor singleton was presented with a higher probability (65%) at a specific location (high-probability location; counterbalanced across participants), whereas all other locations had a lower probability of containing the distractor (35%; low-probability location; see also Wang & Theeuwes, 2018a). When the distractor was absent, the target had an equal chance of appearing at any of the eight locations. Participants first practiced the task (40 trials) and then performed six blocks of 120 trials (for a total of 720 trials). Participants were given feedback for incorrect responses or when their response exceeded the time limit (3,000 ms).

## Results

Trials with response times (RTs) exceeding 2,000 ms (3.4%), incorrect responses (4.2%), and a saccadic onset shorter than 80 ms or longer than 600 ms (6.9%; see also Theeuwes & Belopolsky, 2012; van Zoest, Donk, & Theeuwes, 2004) were excluded from further analyses.^1^ Eye movements were classified as saccades if their velocity was greater than 35 °/s and their acceleration greater than 9,500 °/s^2^. Furthermore, we considered an eye movement as a saccade if it exceeded 2° from the fixation cross. Following these inclusion criteria, 13.5% of the data in total were excluded with partial overlap from previous rules. A saccade was marked as landing on the target or the distractor if its endpoint was within 2° of the target or the distractor, respectively.

### Manual reaction times

#### Attentional capture

**Fig. 2:**
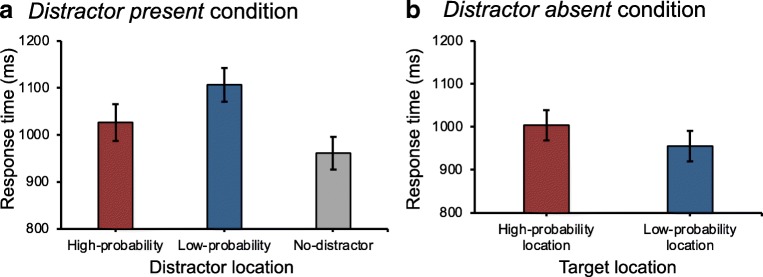
**a** Mean response times (RTs) as a function of distractor location. **b** Mean RTs when the target was presented at the high- vs. low-probability location when no distractor was presented. Error bars represent ±1 the standard error of the mean

We examined the effect of the distractor location on manual RTs (see Fig. 2a). A repeated measures ANOVA on mean RTs with distractor condition (no-distractor, low-probability location, and high-probability location) as a factor revealed a main effect of distractor condition, *F*(2, 30) = 70.09, *p* < .001, partial *η*^2^ = .82. Planned comparison showed that mean RTs was faster in the no-distractor condition (961 ± 136 ms) compared to the low-probability location (1,107 ± 139 ms), *t*(15) = 9.96, *p* < .001, *d* = 1.06; and similarly compared to the high-probability location (1,027 ± 152 ms), *t*(15) = 6.17, *p* < .001, *d* = 0.46. Crucially, mean RTs was faster in the high-probability location than in the low-probability location, *t*(15) = 7.06, *p* < .001, *d* = 0.55, consistent with the idea of decreased attentional capture for the high-probability location.

#### Efficiency of target selection

We investigated the effect of the target location on manual RTs when the distractor was absent (see Fig. 2b). A paired *t*-test showed that observers responded slower when the target appeared at the high-probability location (1,004 ± 135 ms) than any other location on the display (956 ± 137 ms), *t*(15) = 3.35, *p* = .004, *d* = 0.36, indicating that the efficiency of selecting the target was lower when the target appeared at the high-probability location compared to the low-probability location.

### Analysis of eye movements

#### Landing position of eye movements (distractor singleton present)

**Fig. 3:**
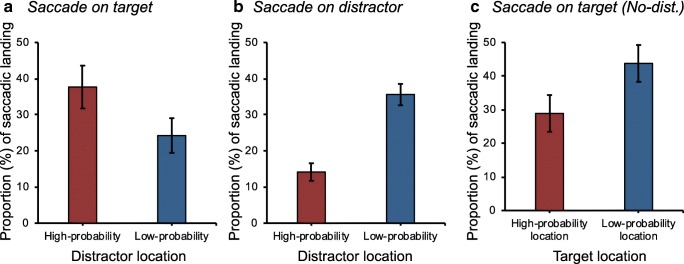
**a** Proportion of first saccades landing on the target as a function of distractor location. **b** Proportion of first saccades landing on the distractor as a function of distractor location. **c** Proportion of first saccades landing on the target when the target is presented at the high- vs. low-probability distractor location when no distractor is present. Error bars represent ±1 the standard error of the mean

We examined the effect of the *distractor location* on the landing of first saccades when they were directed at the target (see Fig. 3a). A paired *t*-test showed that more eye movements landed on the target when the distractor appeared at the high-probability location (37.7 ± 22.9 %) than at the low-probability location (24.3 ± 18.6 %), *t*(15) = 5.96, *p* < .001, *d* = .64. This suggests that distractors displayed at the high-probability location competed less for attention than distractors displayed at the low-probability location.

Moreover, to further examine the effect of *distractor location* on the direction of the first eye movement, we assessed how often the initial eye movement was directed at the distractor location depending on whether the distractor appeared at a high- versus a low-probability location (see Fig. 3b). Suppression of the high-probability distractor location would result in fewer saccades landing on the distractor when it appeared in the high-probability location than in the low-probability location. A paired *t*-test demonstrated that fewer saccades landed on the distractor location when the distractor appeared at the high-probability location (14.2 ± 9.4 %) than at the low-probability locations (35.6 ± 11.5 %), *t*(15) = 5.97, *p* < .001, *d* = 2.05). This finding suggests that oculomotor capture by the distractor was decreased for the high- compared to the low-probability location.

#### Landing position of eye movements (distractor singleton absent)

We examined the effect of *distractor location* on the landing of first saccades by examining only trials in which the distractor singleton was absent (see Fig. 3c). A paired *t*-test showed that fewer first saccades landed on the target when it appeared at the high-probability location (28.9 ± 21.0 %) than at the low-probability location (43.8 ± 21.1 %), *t*(15) = 3.39, *p* = .004, *d* = 0.71. This suggests that observers were less efficient in selecting the target when it appeared at the high-probability location than at any other location even when no distractor was present.

#### Saccadic latencies for saccades to the distractor and the target

We examined the effect of *distractor location* on the onset of saccadic eye movements towards the distractor. There was no significant difference between the saccadic latencies when the distractor appeared in the high- (242.7 ± 44.6 ms) compared to the low-probability location (232.6 ± 55.5 ms), *t*(15) = 1.63, *p* = .125, *d* = 0.20.

**Fig. 4:**
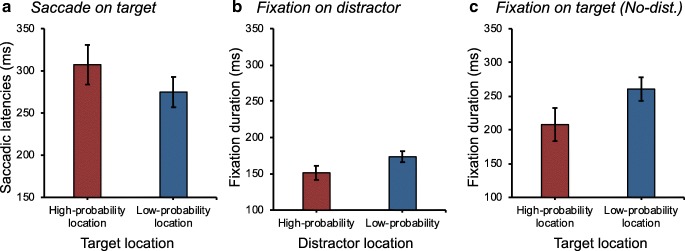
**a** Saccadic latencies to the target when the target was presented at the low- vs. high-probability distractor location when no distractor is present. **b** Fixation duration on the distractor as a function of distractor location. **c** Fixation duration on the target when the target was presented at the low- vs. high-probability distractor location when no distractor is present. Error bars represent ±1 the standard error of the mean

We also examined saccade latencies towards the target when it happened to be at a high- versus a low-probability location in the distractor singleton absent trials (see Fig. 4a). If the high-probability distractor location is suppressed compared to all other locations we would expect saccades to be initiated slower towards the high-probability location than to any of the other locations. Indeed, a paired *t*-test showed that saccades were initiated slower when the target appeared at the high-probability location (307.3 ± 90.8 ms) than at any other location (274.8 ± 69.3 ms), *t*(15) = 3.35, *p* = .004, *d* = 0.40, once more supporting the notion that the high-probability distractor location was suppressed compared to other locations.

#### Attentional dwell

We examined whether *distractor location* affected fixation duration when the eye movements landed on the distractor in distractor singleton present condition (see Fig. 4b), and on the target in distractor singleton absent condition (see Fig. 4c). If the reduced interference effect is also due to rapid disengagement from the high-probability location, it is expected that fixations (dwell times) should be shorter for distractors and targets at the high-probability location than at the low-probability location. A paired *t*-test revealed that fixations on the distractor were indeed shorter when it appeared at the high-probability location (151.3 ± 37.1 ms) than at the low-probability location (173.6 ± 29.3 ms), *t*(15) = 5.22, *p* < .001, *d* = 0.67. Also, fixations on the target were indeed shorter when it appeared at the high-probability location (208.0 ± 94.9 ms) than at the low-probability location (260.6 ± 67.6 ms), *t*(15) = 2.91, *p* = .011, *d* = 0.64. Together, this supports the rapid disengagement hypothesis.

### Inter-trial repetition effect

To examine whether the current findings are the result of transient inter-trial location repetition effects, we excluded those trials on which the distractor was successively presented at the high-probability location (i.e., high- to high-probability location). This analysis allows us to check whether the main findings still hold. When those trials were removed, the analyses showed for all dependent measures there was still a difference between the high probability versus the low probability location: for manual RTs (1,029.1 ms vs. 1,108.2 ms), for the proportion of saccades landing on the distractor (15.1% vs. 37.4%), and for the fixation duration when the eye movements landed on the distractor (157.2 ms vs. 174.1 ms); all *t*s > 2.99, all *p*s < .009. The results indicate that the current findings cannot be explained by inter-trial location repetition.

### Time course of the effect

#### Manual RTs

To examine the time course of the suppression effect on manual RTs, the data were submitted to a repeated measures ANOVA on RTs with *block number* (1–6) and *distractor condition* (no distractor, low-probability, and high-probability location) as factors, revealing a significant main effect of block, *F*(5, 75) = 47.19, *p* < .001, partial *η*^2^ = .76 and distractor location, *F*(2, 30) = 71.03, *p* < .001, partial *η*^2^ = .83. The interaction between block and distractor location was significant, *F*(10, 150) = 2.60, *p* = .006, partial *η*^2^ = .15. However, subsequent planned comparison showed that the main effect existed in each block, all *F*s > 23.93, all *p*s < .001, suggesting that the suppression effect on manual RTs did exist over blocks.

#### Landing position of eye movements on distractor

With the same analysis as manual RTs, a repeated measures ANOVA on the proportion of landing position on distractor with block (1–6) and distractor condition (low-probability vs. high-probability location) as factors revealed a significant main effect of distractor location, *F*(1, 15) = 31.49, *p* < .001, partial *η*^2^ = .68, but not for block, *F*(5, 75) = 1.66, *p* = .155, partial *η*^2^ = .10. The interaction between block and distractor location was not reliable, *F*(5, 75) = 1.57, *p* = .18, partial *η*^2^ = .10, suggesting that the reduced oculomotor capture effect did not change over time.

#### Attentional dwell

With the same analysis as manual RTs, a repeated measures ANOVA on fixation duration when the eye movements landed on the distractor with block (1–6) and distractor condition (low-probability vs. high-probability location) as factors revealed a significant main effect of distractor location, *F*(1, 15) = 12.02, *p* = .003, partial *η*^2^ = .45, but not for block, *F*(5, 75) = 1.44, *p* = .22, partial *η*^2^ = .09. The interaction between block and distractor location was not reliable, *F*(5, 75) = 0.98, *p* = .439, partial *η*^2^ = .06, suggesting that the disengagement effect did not change over time.

### Assessment of observers’ awareness of the statistical regularities

To examine if observers noticed the statistical regularities, we asked observers to complete a questionnaire after the experiment was concluded that contained a depiction of eight circles placed on the radius of an imaginary circle. Observers had to report whether they thought the color singleton distractor was presented more often at a specific location and, if so, to mark that location. Furthermore, they were asked to indicate their level of certainty regarding their decision on a scale of 1–7 (7 being the highest). Out of 16 observers, ten indicated that they did not think that the distractor was displayed with a higher probability in a specific location. Out of the remaining six observers who indicated that they thought the distractor appeared at a specific location more often, two indicated the correct location (i.e., the high-probability distractor location). The confidence mean score for the participants that indicated the correct location was 3.0, suggesting that their confidences were low.

## Discussion

The present study investigated whether the reduced interference of distractors appearing at high probability distractor locations is the result of spatial suppression or rapid disengagement. Our results show that both effects play a role. On the one hand, there is clear evidence for spatial suppression of high likelihood distractor locations. Observers made fewer saccades to the more likely distractor location, and if the target was displayed at the high-probability distractor location, they made fewer first saccades to that location and were also slower in initiating them. Additionally, observers made more saccades directly to the target when the distractor was at the high-probability distractor location. On the other hand, however, there is also evidence for rapid disengagement, as fixation durations on the distractor were shorter when it appeared at the high- compared to the low-probability distractor location. It is assumed that after a saccade is made to a location, observers process the information presented at that location, whereby if the location contains the target, observers emit a response or otherwise disengage from the location. The current findings suggest that if a distractor is displayed in the high-probability distractor location and observers made a saccade to it, they also disengaged faster from that location relative to when the distractor was displayed at any other location. This may imply that observers learned that the target was less likely to appear at the high-probability distractor location. Even though it is clear that there is faster disengagement from the high- compared to the low-probability distractor location, this effect was relatively small (22.3 ms), and as such it cannot account for the relatively large reduction in RT interference as found in the current study and previous attentional studies (Wang & Theeuwes, 2018a, 2018b). Therefore, our findings suggest that both effects (i.e., suppression of the high-probability distractor location and faster disengagement from that location) play a role in the observed reduced interference effects due to SL, with a much larger role of suppression than for faster attentional and oculomotor disengagement.

Our findings are consistent with those of Wang and Theeuwes (2018a, 2018b). They employed a covert attention task demonstrated that when the distractor appears at a high-probability location it results in less RT interference than when it is presented at any other location (Wang & Theeuwes, 2018a; for similar results see Ferrante et al., 2018; Goschy et al., 2014). These findings were explained by assuming that observers implicitly learn the embedded statistical regularities, resulting in the suppression of the more likely distractor location. The current findings provide compelling evidence that the high-probability distractor location is, indeed, suppressed compared to any other location. Because of the suppression of the high-probability distractor location, we saw that (1) observers made fewer saccades to this location, (2) if the target appeared at this location observers were slower to initiate a saccade, and (3) observers made more saccades directed to the target when the distractor was presented at the high-probability distractor location than when it was presented at any other location. This pattern of results is consistent with the notion that through SL, the weights in the attentional selection priority map are adjusted, resulting in the high-probability distractor location competing less for attention compared to other locations (e.g., Ferrante et al., 2018; Wang & Theeuwes, 2018a). Note, however, that the current findings suggest that an additional mechanism referred to as speeded disengagement may also play a role in reducing interference effects. We show a small effect of faster disengagement from the high- compared to the low-probability distractor location (see also Brockmole & Boot, 2009). This effect may also play a role in search tasks in which observers do not make saccades (as in Wang & Theeuwes, 2018a, 2018b); nevertheless, this would then be seen in faster covert disengagement of attention from the high-probability distractor location.

A recent eye-tracking study (Gaspelin, Leonard, & Luck, 2017) examined distractor suppression in a visual search task, in which observers were encouraged to search for a particular shape (so-called "feature-search" mode; Bacon & Egeth, 1994). They found that only when observers were instructed to search for a target based on a specific feature (Experiment 2) instead of a singleton (Experiment 1), were the salient distractors almost perfectly suppressed relative to the non-salient distractors, as evidenced by fewer first saccades landing on the salient distractor relative to the non-salient distractors. These findings were interpreted as supporting the "signal suppression" hypothesis, which suggests that salient stimuli engender a bottom-up signal that can then be inhibited by means of top-down processes (Sawaki & Luck, 2010; see also Gaspelin, Leonard, & Luck, 2015; Gaspelin et al., 2017).

Similar to Gaspelin et al. (2017), we also report fewer first saccades landing on the suppressed location providing converging evidence that suppression is manifested in the oculomotor system. There are, however, also important methodological differences between Gaspelin et al.’s (2017) and our study. First, we employed the additional singleton paradigm, where participants searched for a shape singleton. It has been consistently shown that in the additional singleton paradigm, saccades are captured by the singleton distractor (e.g., Experiment 1 in Gaspelin et al., 2017; see also Theeuwes et al., 2003; Theeuwes, 1991), and, indeed, our results showed consistent capture by the distractor in each possible location. Crucially, however, our task contained embedded regularities, whereby the distractor was presented more often at one location than at any other location on the display. As expected, despite a consistent capture effect for all locations, we found differences in the extent of capture when comparing the different probability conditions: capture was decreased when the distractor was displayed at the high-probability distractor location compared to any other location on the display (as in Wang & Theeuwes, 2018a, 2018b). So, Gaspelin et al. (2017) showed that in a task promoting feature-search, oculomotor capture by the salient distractor is reduced, while we showed decreased oculomotor capture as a result of statistical learning.

It has been argued that oculomotor (and attentional) capture is driven by bottom-up salience; however, the subsequent oculomotor or attentional disengagement from a given location is considered to be top-down in nature (Born et al., 2011; Godijn & Theeuwes, 2002; Mulckhuyse et al., 2009; Schreij et al., 2010; Theeuwes, 2010; Theeuwes et al., 2003). Since we observed faster disengagement from the high-probability location compared to the low-probability location, one may assume that this faster disengagement is also top-down. Even though possible, it is not likely that this faster disengagement is volitional in origin. To actively suppress the high-probability distractor location, it would require that observers are aware of the distractor being presented more often in that location (see Theeuwes (2018) for a recent discussion on the topic of volition and top-down control). Since most of the observers could not accurately report the high-probability distractor location, it is improbable that they volitionally suppressed it. This would suggest that rapid disengagement might not only rely on top-down mechanisms, but can also emerge as a result of implicit statistical learning.

In sum, our results demonstrate that statistical regularities regarding the distractor affect oculomotor control. We found reduced oculomotor capture for the high-probability distractor location compared with all other locations. We assume that through statistical learning the more likely distractor location competes less for attention (and eye movements) in the spatial priority map than all other locations.

## Footnotes

1 The use of a relative RT cutoff (i.e., 2.5 SD strandard) did not change any of the results patterns.
